# Hyperglycosylated gp120 mutants elicit improved CD4-binding site directed antibodies in a heterologous prime:boost regimen

**DOI:** 10.1186/1742-4690-9-S2-P73

**Published:** 2012-09-13

**Authors:** FK Ahmed, BE Clark, R Pantophlet

**Affiliations:** 1 Simon Fraser University, New Westminster, Canada; 2 StemCell Technologies, Vancouver, Canada

## Background

The CD4-binding site (CD4bs) on gp120 is targeted by broadly neutralizing antibodies (nAbs) and is therefore of interest for vaccine design. Insight derived from molecular interactions of CD4bs-specific antibodies with gp120 has guided structure-based protein design and the development of a number of immunogens. Of equal interest are strategies to improve potency and durability of desired nAb responses to such immunogens.

## Methods

We generated truncated, hyperglycosylated gp120 mutants designed to selectively present nAb epitopes overlapping the CD4bs. To help focus antibody responses to these epitopes, we conducted a heterologous prime:boost immunization using two mutants (termed ΔN2mCHO and ΔN2mCHO(Q105N)) in combination with a resurfaced gp120 core protein (RSC3) that preferentially presents the CD4bs neutralizing face. Groups of animals were primed with ΔN2mCHO, unappended or N-terminally appended with one of three immunostimulatory sequences known to amplify humoral responses – PADRE, N10 or C3d. The animals were boosted with RSC3 and then ΔN2mCHO(Q105N). Serum specificities were dissected using CD4bs and non-CD4bs mAbs and responses were followed at the cellular level by phenotyping the memory B cell compartment after each injection.

## Results

Relative to other groups, PADRE-ΔN2mCHO elicited significantly more rapid and higher titres against gp120 and the immunogens, suggesting that PADRE has superior immunoactivating properties. The PADRE- antibodies also bound greatest to epitopes overlapping the CD4bs. Unexpectedly, only sera from N10- animals exhibited significant neutralizing activity against select tier 1B and 2 viruses. Unanticipatedly also, no significant differences were observed at the memory B cell level between the groups for gp120 specificity.

## Conclusion

Our results show that selective exposure of conserved epitopes through the use of varied immunogens fused with immunostimulatory sequences, in particular PADRE, can boost desired antibody responses. Together, our data highlight the importance of not only immunogen design but also formulation on directing antibody responses to conserved epitopes.

